# Retracted: Prenatal Diagnosis of Concurrent Achondroplasia and Klinefelter Syndrome

**DOI:** 10.1155/2016/4363897

**Published:** 2016-04-28

**Authors:** Case Reports in Obstetrics and Gynecology

The article titled “Prenatal Diagnosis of Concurrent Achondroplasia and Klinefelter Syndrome” [1] has been retracted as the same case report was found to have been presented in the following previously published article: “Achondroplasia with 47, xxy karyotype: A case report of the neonatal diagnosis of an extremely unusual association,” BMC Pediatrics 2012, 12:88. The article has also been published without the consent of Dr. Cristina Martinez-Payo. The first author, Dr. Esther Perez-Carbajo, assumes full responsibility.
